# Environmental Exposure: Effect on Maternal Morbidity and Mortality and Neonatal Health

**DOI:** 10.7759/cureus.38548

**Published:** 2023-05-04

**Authors:** Usha Kumari, Raj Kishor Sharma, J R Keshari, Archana Sinha

**Affiliations:** 1 Biochemistry, Indira Gandhi Institute of Medical Sciences, Patna, IND; 2 Microbiology, Patna Medical College, Patna, IND; 3 Biochemistery, Indira Gandhi Institute of Medical Sciences, Patna, IND; 4 Obstetrics and Gynaecology, Indira Gandhi Institute of Medical Sciences, Patna, IND

**Keywords:** mortality, morbidity, pollution, environmental exposure, child health, maternal health

## Abstract

Environmental factors are important causes that impair global pregnancy outcomes and are, importantly, responsible for maternal morbidity and mortality. However, apart from the direct reasons for maternal deaths, mainly obstetric and neonatal complications, such factors are ignored or given less importance. The recent surge in research on the impact of various environmental factors on pregnancy outcomes suggests the need for immediate attention to such factors and device-specific policies to counter the situation. Moreover, the recent coronavirus disease of 2019 (COVID-19) pandemic, global warming, and climate change showed a lack of preparedness to counter the impact of such events on maternal survival and safe and successful pregnancy outcomes. In the present review, we have emphasized the specific factors responsible for increased maternal and neonatal deaths and their association with specific environmental factors. Increased attention on maternal healthcare, preparedness to counter sudden environmental challenges and improvement of the conventional requirement for better maternal healthcare access and nutrition at a global level may improve the scenario.

## Introduction and background

The complications of maternal morbidity and mortality and their association with neonatal health have been a global problem for a long time. However, the most affected countries related to maternal morbidity, mortality, and neonatal health are the middle- and low-income countries [1].

Socio-economic conditions, access to affordable and better-quality healthcare facilities, education level, and other demographic and clinical factors were found to be responsible for such conditions. Recent surveys suggest that a global integrated effort by medical and social welfare organizations and local governments was successful in reducing maternal and newborn deaths by almost half in number between 1990 and 2020. The global estimation of UNICEF suggests the death of 2.4 million in the year 2020 alone; further, the estimated daily death due to pregnancy complications is 810 globally [2]. However, unacceptably high numbers of babies and mothers, particularly young moms, are still dying, largely from preventable or treatable reasons such as infectious infections and problems during pregnancy or childbirth. In recent years, we have seen alarming setbacks for women's health, with maternal fatalities increasing or remaining stable in practically every part of the world. Many countries struggle to improve mother and newborn survival and reduce stillbirths due to inequitable access to inexpensive, high-quality health care and services. A large proportion of maternal and neonatal mortality occurs in conflict or displacement contexts. If current trends continue, 48 million children under the age of five will die between 2020 and 2030, with half of them being newborns. The major causes of maternal mortality are severe bleeding, high blood pressure, pregnancy-related illnesses, and complications from botched abortions. All of these are generally avoidable with access to high-quality healthcare. If this trend continues, the lives of almost 1 million additional women will be jeopardized by 2030 [2,3]. Hence, such data suggest the required immediate requirement of global attention to improve the situation irrespective of the demographical factors.

## Review

Global status of neonatal health, maternal morbidity and mortality burden

Several reports have provided ample data on the global scenario related to maternal health conditions, neonatal health, and associated risk factors, along with their possible solutions, for a long time [3-6]. Estimation and evaluation of the maternal mortality trend at the global level showed improvement in the situation; however, at the country level, contradictory and diverse outcomes were observed for densely populated larger countries [5]. At the economic level, the global burden of severe maternal mortality (SMM) ratio was reported to be higher in low- and middle-income countries (LMICs) compared to high-income countries [6]. In an African country, such as Nigeria, a region-specific strategy has been recommended to reduce the incidences [7]. Further, the strategically implemented health extension programme (HEP) and development of the health development army (HAD) programme reflected a significant and drastic reduction in the maternal mortality ratio (MMR) in Ethiopia [8]. Similar to the African nations, other South Asian countries also reported the impact of huge maternal mortality and morbidities, along with cases of newborn mortalities [9,10].

The burden of maternal morbidity and mortality in Indian states

The MMR declined in India by about 70% from 398/100,000 live births (95% CI 378-417) in 1997-98 to 99/100,000 (90-108) in 2020. About 1.30 million (95% CI 1.26-1.35 million) maternal deaths occurred between 1997 and 2020, with about 23,800 (95% CI 21,700-26,000) in 2020, with most occurring in poorer states (63%) and among women aged 20-29 years (58%). The MMRs for Assam (215), Uttar Pradesh/Uttarakhand (192), and Madhya Pradesh/Chhattisgarh (170) were the highest, surpassing India’s 2016-2018 estimate of 113 (95% CI 103-123) [11].

At present, according to socio-economic surveys, Bihar is considered one of the lagging states. An improvement from 18.7% to 34.6% in antenatal health check-up percentage was observed in the National Family Health Survey (NFHS) and NFHS4 in Bihar; however, this percentage was quite lower than the survey result in Madhya Pradesh [12,13]. The latest NFHS5 survey (2019-2020) highlighted that antenatal check-ups in the first trimester were done by 59.8% of pregnant women in urban areas and 51.9% in rural areas [14].

Several scientific investigations explored the plausible reasons for higher MMR and newborn mortalities globally, especially for African nations and South Asian countries, along with the other LMICs. It was observed that varying causes are responsible, apart from the already discovered association of socio-economic factors. With ongoing climate change and ever-changing global environmental scenarios, different environmental causes are being detected as associated reasons for higher maternal mortality and morbidity rates and newborn mortality. Such environmental exposures encompass socio-economic conditions, behavioural factors, cultural conditions of the neighbourhood, and close social interactions between the mother and the child [15].

In the present article, we focused on important environmental factors that impact mother and child health, along with global statistics on maternal and newborn morbidity and mortality.

Disparity in environmental causes for maternal health

The global improvement in the maternal mortality ratio has been witnessed in the last 5 years; however, there is still immense scope for improvement in maternal and child health conditions for better outcomes. Unfortunately, at the global level, a disparity has been observed in maternal and newborn healthcare conditions, which drastically vary from country to country or even in a country with varied socio-economic strata. Interstate variation is very alarming in India [11]. Hence, the best possible solution could be better and unrestricted access to the best possible reproductive healthcare facilities for all. Global analyses and reports suggest many direct and indirect causes that are associated with poor maternal health care conditions. Older reports suggest that the majority of the maternal deaths in Asia, Africa, and some European countries were associated with direct obstetric reasons such as sepsis, haemorrhage, infections such as hepatitis due to poor sanitization and hygienic conditions, treatment complications, complications related to hypertension, ruptured uterus, malnutrition, anaemia, and others [16,17].

Direct Impact

Managing pregnancy throughout the gestational period requires utmost maternal care, not only for the health of the mother but also for the future child's health. Such a level of care warrants the requirement of easier and faster access to basic healthcare facilities, the presence of doctors, nurses, and trained staff, along with logical and scientific decision-making capabilities both for the family and the medical practitioners. However, certain situations involving maternal conditions require immediate attention. Specific conditions that may impact maternal and child health, morbidity, and mortality include hypertension, gestational hypertension, preeclampsia, especially in the first trimester, and gestational diabetes [18-20]. Genetic, epigenetic, and inheritable complications can also modulate certain disease conditions, such as gestational diabetes mellitus (GDM). Differential gene expression patterns have been identified in the gravid and non-gravid populations through genome-wide association studies, where expression of the GDM-causing genes was observed in the gravid population [21].

Indirect Impact

Several socio-economic and environmental factors play a crucial role in pregnancy management and maternal and child healthcare. Several indirect obstetric reasons also influence maternal deaths. Indirect obstetric reasons include previously existing diseases or diseases that may develop during pregnancy due to inheritable and acquired disease conditions in pregnant women. Indirect reasons for maternal death also include the lack of facilities in emergency obstetric care (EmOC) during pregnancy management and delivery [22]. Say et al. reported that a considerable percentage (27.5%) of maternal deaths occurred globally due to the influence of indirect causes [23]. Communicable and non-communicable diseases play a pivotal role in increasing the maternal death rate globally, especially due to a lack of maternal healthcare and disease management. Pre-existing medical conditions account for the majority (14.8%) of the indirect global burden of maternal deaths due to non-communicable diseases; however, communicable diseases like HIV (5.5%) and malaria are also responsible for such indirect maternal morbidities [24,25].

Various environmental factors also contribute to pregnancy outcomes. The role of various pollutants, such as air pollution, in reduced fertility and the association of air pollution with female fertility are well studied [26,27]. The association of certain compounds, such as NO_2_ and ozone, with pregnancy outcomes is well documented [28-30].

Complications and changing scenarios in pregnancy

Management of pregnancy and associated healthcare require dedicated, trained personnel with scientific knowledge and experience, as each case differs from the other and the possible complications in different trimesters vary from each other on a case-by-case basis. The dynamic condition remains challenging to the healthcare provider and medical practitioners and requires keen attention, understanding, and observation of the ongoing pregnancy, along with the proper and timely diagnosis and therapeutics as required. Depending on the case, a successful pregnancy outcome requires the management of varying complexities associated with pregnancy, close monitoring, the supply of medical and healthcare facilities, effective decision-making by the medical practitioner and the family, and constant support from the socio-economic condition. However, the dynamic condition may face various complications and challenges from time to time and require timely resolution on a case-by-case basis.

Medical conditions and common risk factors associated with pregnancy

The demographic conditions, such as age and associated factors, and medical conditions, such as general health conditions and pre-existing diseases, of pregnant women affect pregnancy and the outcomes. Hence, assessment of the health condition and evaluation of the risk factors are mandatory for every pregnancy to ensure a safe and successful outcome and to avoid complications.

Various risk factors are reported that are commonly associated with different stages of pregnancy and are managed through proper medical and therapeutic interventions as required. Often, medical practitioners categorize pregnancy based on the risk factors associated with a high-risk, medium-risk, or low-risk pregnancy, and decisions on the treatment course are made accordingly for a safe and successful outcome. High-risk pregnancy includes advanced maternal age (>35 years), unhealthy lifestyle of the mother such as smoking, drinking over the permissible limits, illegal use of drugs and banned substances, pre-existing severe disease conditions, multiple pregnancy conditions, complications during the pregnancy terms such as uncommon placental position, restricted or impaired foetal growth than the estimated normal growth during pregnancy, and rhesus factor complications (rh+ or rh−) [31]. Apart from the mentioned complications and risk factors, numerous studies were conducted in specific contexts related to pregnancy-associated risk factors. Thompson et al. reported that in a specific socio-economic condition, homeless teens with pregnancy, i.e., 20% of the total homeless population, even suffer for proper shelter during pregnancy in a developed country like the USA [32]. Hence, the associated risk factors for pregnancy and outcomes are complex and encompass health and medical, socio-economic, demographic, and other factors. Certain common and important risk factors associated with pregnancy complications are discussed here.

Pregnancy and Anaemia

Due to socio-economic or nutritional deficiency, genetic, or pregnancy-associated physiological alterations, anaemia is observed in many pregnant women at various stages of pregnancy. Pregnancy-associated anaemia is reported in many populations during pregnancy, especially in Asian countries. In China, the prevalence of maternal anaemia reported was 23.5% of the total pregnancy and was found to be significantly associated with the growing maternal age, per-capita income of the family, and other factors [33,34]. In India, the prevalence of anaemia in pregnant women (15-49 years) is 52.2%. There has been an increase in the prevalence of anaemia as per NFHS 5 compared to NFHS 4 [12-14]. Evaluation of the adverse outcomes suggested more NICU admissions after the births, pre-term deliveries, neonatal complications, and other adverse medical conditions [33]. Certain environmental conditions, such as poor socio-economic and educational background, lack of nutrition and proper diet during pregnancy, and deficiency of healthcare facilities, especially blood banks and iron supplement-associated therapies, are the major causes of pregnancy-associated anaemia, especially in sub-Saharan African regions [35]. Similar reports were available from other LMIC countries such as Nigeria, Tanzania, and others [36-38].

Pregnancy and Hypertension

Hypertension is a common problem in a large global population. However, a recent report suggested that the prevalence of hypertension is higher in the adults of the LMIC countries (31.5%) than in the adult population of the developed countries (28.5%) [39]. Similarly, acute and chronic hypertension during pregnancy are reported in different studies [40]. Such hypertensive conditions can induce cardiovascular risk in pregnant women as a long-term effect due to augmented cardiovascular ageing and the onset of valvular heart complications in pregnant women. Further, the presence of chronic hypertension during pregnancy may result in an impaired normal delivery, preeclampsia, impaired growth of the foetus, preterm delivery, and also perinatal and maternal death [41].

Many associated factors were reported with pregnancy and hypertension, including preeclampsia, caesarean delivery, cerebrovascular accidents, foetal growth restriction, preterm birth, and maternal and perinatal deaths. Environmental factors, such as toxicants, were reported to be associated with hypertension in pregnancy. Even though inconclusive, specific toxicants such as polycyclic biphenyls, phthalates, cadmium, lead, and pesticides were reported to have an association with preeclampsia and reasons associated with hypertension during pregnancy [42].

Metabolic Disorders During Pregnancy

The physiological alterations during pregnancy support the increased metabolic demands of the mother and the foetus. Changes in specific metabolism, such as the metabolism of homocysteine, alteration in general nutrient metabolism, altered drug metabolism, and hormonal regulation, were reported earlier [43-45]. However, most of the metabolic changes are reversible and can return to normal after the completion of the pregnancy. In this process of metabolic changes, several metabolic disorders like endothelial dysfunction, gestational diabetes, calcium metabolism disorder, parathyroid dysfunction, and other metabolic complications may occur in the pregnant mother [46-48]. This kind of metabolic problem can also affect foetal development and child growth. The role of various factors such as perfluoroalkyl substances (PFAS), polystyrene microplastics, and natural and synthetic endocrine disrupting chemicals (EDCs) was found to be harmful to the pregnant mother and the foetus and directly impair placental development, child development, maternal metabolism, and foetal metabolism [49-51].

Role of Genetic and Epigenetic Factors

Genetic factors are directly associated with the health conditions and pregnancy terms of the mother and the foetus. The foetal metabolic condition was reported to be programmed based on maternal obesity and overnutrition associated with the epigenetic control of the metabolic condition of the pregnant mother [52]. Further, diet can influence epigenetic changes that may in turn adversely affect the intrauterine environment [53]. Such altered epigenetic changes and changed gene expression can impact foetal developmental programming and may cause permanent structural changes to the foetal tissues and organs [53]. An association of epigenetic factors is also reported with asthma, nutrition, and maternal diabetes [54-56]. A study reported that smoking during pregnancy can induce epigenetic modification of brain-derived neurotrophic factor 6 in the offspring [57]. Recent studies also reported maternal diabetes in pregnancy [56], and oxidative stress can have a tremendous impact on the child’s epigenome and can induce early vascular diseases in the foetus [58]. Hence, the genetic and epigenetic condition of the mother directly impacts the genetic and epigenetic condition of the foetus. Environmental factors play a crucial role in the proper, healthy development of the foetus and the child. A proper maternal diet can influence the role of epigenetics in protecting the child against environmental pollution [59]. The complex interplay of genetic, environmental, and epigenetic factors was reported to be associated with autism as well [60]. Therefore, it is important to understand the complex interplay and association of the genetic and epigenetic factors concerning safe and successful pregnancy outcomes and their relationship with environmental influencers.

Maternal and neonatal physiology and climate change

Many scientific reports suggest the association of various climatic and geological factors with pregnancy outcomes as part of the environmental causes. Such studies are essential considering the present context of global warming, climate change, and pandemics. In association with the climatic factors, identification of the vulnerable population, evaluation of maternal and child health, and mortality and morbidity are crucial [61].

Environmental conditions

Extreme Events

Extreme and harsh weather and climatic conditions affect pregnancy conditions adversely. In this context, increased frequency of drought, floods, and extreme environmental conditions negatively influence the basic nutritional and healthcare needs of pregnant women in a population that results in poor pregnancy outcomes and higher maternal and foetal mortality. Such statistics are witnessed in populations with poor socio-economic backgrounds and populations impacted heavily by natural calamities [61]. Many sudden impacts on the pregnant condition are witnessed, such as spontaneous abortion, premature contractions, dehydration, low birth weight, malnutrition of the mother and lack of food security, endemic diseases such as diarrhoea, and others.

Temperature

The adverse impact of high temperatures on pregnancy has been reported in many studies. Rostami et al. recently reported that higher and more adverse temperatures may induce chronic toxoplasmosis (CT) in pregnant women [62]. A study on the impact of temperature shocks suggested that higher temperatures may affect parental characteristics and behaviours [63]. A Spanish study suggested a relationship between maternal group B streptococci (GBS) colonization and the increasing climatic temperature that may further impact the condition of the onset of neonatal sepsis [64]. Studies associated with ambient temperature ("heat island effect") for urban pregnant women and congenital heart disease (CHD) suggested no significant association; however, the researchers have identified specific exceptions [65]. Studies considering temperature as a major climatic influencer for pregnancy outcomes establish the facts that changing climatic conditions and increasing global warming should be considered concerns associated with maternal and foetal health and pregnancy outcomes. However, more studies are required to confirm the specific influences and relations between temperature and pregnancy outcomes.

Pollution

The adverse effect of pollution on human health is well-researched and a scientific fact. Ample research has been conducted to understand the various effects of pollution, especially air pollution, water pollution, and industrial pollution [66].

Air pollution: The impact of air pollution on hypertension-associated pregnancy, polycystic ovarian disorder, reduced fertility, and miscarriage is well documented [67,68]. In the case of foetus development and neonatal birth, lower birth weight and affected lung development of the foetus are some of the adverse effects of air pollution [69]. The association between ambient air pollution and hypertensive disorder (HDP) in pregnant women has been well investigated by many studies and established [70-72].

The association of PM10 and NO_2_ with systolic blood pressure in the different trimesters of pregnancy was observed in the pregnant women's population of the Netherlands [70]. However, a significant relationship with pregnancy-induced hypertension was seen only for the PM10 level in this study. In another study, Hu et al. established that constant NO_2_ exposure during pregnancy can cause pregnancy-associated HDP [71]. Similar results were reported by Mobasher et al.: an association between PM2.5 and carbon monoxide showed increased HPD in the first trimester, and ozone (O_3_) was found to be adversely effective in the second trimester [72]. In another cohort study, it was reported that in the first and second trimesters of pregnancy, exposure to NO_2_, O_3_, PM2.5, and PM10 can enhance the risk for preeclampsia, whereas in the second trimester of pregnancy, increased levels of SO_2_ and CO exposure may become the major risk factor for pre-eclampsia [73]. Exposure to high-level air pollution can induce the development of HDP at an early stage of pregnancy [68].

A significant association between particulate matter (PM2.5) and blood pressure parameters was reported recently [74]. Short-term and long-term exposure to particulate matter (PM2.5) can alter blood pressure-related factors, as evidenced by analyzing the data of residents with varying exposure to higher ambient (PM2.5) air quality. Increased exposure to ambient air (PM2.5) enhances the hospital admission of patients due to hypertension, especially cardiovascular (CVD) patients and pregnant women with elevated blood pressure-related complications [74]. Vasoconstriction occurs due to regular exposure to such ambient air that changes the normal hemodynamics of the patient; further, enhanced inflammation and raised prooxidative mediators were also reported due to the continuous exposure to ambient air (PM2.5). In a recent study, prenatal maternal stress was also reported to be associated with ambient air pollution [75].

All these reports were successful in establishing the relationship between hypertensive disorder during pregnancy, impaired neonatal development, and air pollution; hence, the environmental factors are crucial for pregnancy management and outcomes.

Water pollution: Water pollutants are often considered severely harmful to pregnant women and their foetuses. Certain pollutants such as arsenic, uranium, lead, trihalomethane, hexavalent chromium, cadmium, and nitrate are highly hazardous to the health of pregnant women and the foetus. In California, such water pollutants were found to be tremendously influential in increasing hypertensive disorders in pregnancy [76]. An earlier study conducted in the same region on pregnant women to understand the exposure to trichloroethane through drinking water also reported adverse outcomes [77]. In Punjab, India, heavy metals and pesticides present in the drinking water were recommended as potential risk factors for possible adverse pregnancy outcomes [78]. Similarly, high-concentration arsenic exposure through drinking water was found to be associated with higher stillbirth rates in West Bengal [79]. Further, exposure to a higher concentration of fluoride through drinking water was reported to be responsible for the lower mean concentration of vitamin D (<10 ng/ml) in pregnant women irrespective of their access to a regular, adequate diet and other sources of vitamin D; hence, fluoride exposure through water may become hazardous and adversely effective for the pregnancy outcomes [80].

Occupational exposure and pregnancy outcomes

Occupational health hazards are a global concern, especially for low-income group members and labourers. Developing and third-world countries require special attention to understand the plausible cause and prevention of several occupational hazards involving a major part of the underprivileged population. In India, reports are available on the exposure to municipal solid waste, musculoskeletal problems of the child labourers in the brickfield, exposure to lead, etc. [81-83]. Most of these occupational hazardous exposures are caused by the population belonging to economically poor backgrounds and consisting of workers of both genders and children. Hence, pregnant women are often exposed to radioactive or non-radioactive hazardous substances that may affect pregnancy outcomes.

Exposure to Lead

In India, apart from various occupations in the unorganized sector, possible exposure to lead may occur in the cosmetic and traditional medicine-related industries [84]. The adverse and toxic effect of lead on the human reproductive system has already been reported [85]. Apart from occupational aspects, lead exposure may occur in a normal household through paints and children's polyvinyl chloride (PVC) toys [86]. Impaired childhood executive function and behaviour were observed due to continuous lead exposure [87]. High levels of lead exposure (>40 µg/dl) for short or long durations (>25 µg/dl for over a year) and lead levels >10 µg/dl in maternal blood enhance the possibility of spontaneous abortion, preterm delivery, low foetal weight during birth, and impaired foetal neurological development [88].

Migration of population and socio-economic background

Among demographic causes, migration is an important factor in pregnancy outcomes. Healthy migration due to personal or professional reasons with proper healthcare management during pregnancy may not affect pregnancy outcomes much; however, forced and unprepared migration during pregnancy may have an adverse and severe outcome that may lead to maternal and neonatal death as well [89]. Kragelund et al. recently reported the presence of GDM in the immigrant pregnant population; however, the study concluded that the country of origin of the immigrant and the ambiance they are coming from may be responsible for such adverse pregnancy outcomes and associated complications [90]. Gibson-Helm et al. suggested that significant differences in pregnancy management and outcomes were observed between the migrant women from the humanitarian source countries and the non-humanitarian source countries [91]. Therefore, socioeconomic background remains a determining factor for the pregnancy outcomes of migrant women. However, there are individual causes that may vary before and after migration and may tremendously impact the pregnancy outcome on a case-by-case basis. Apart from the direct adverse outcome-inducing causes, all indirect factors of the safe and successful pregnancy outcomes may depend on the socio-economic background of the family. Socioeconomic background is so important that it may cause major differences even with the availability of a universal healthcare system [92]. Specific socioeconomic conditions such as monthly family income, maternal and paternal education, social status of the family, and paternal occupation directly impact the pregnancy management level and the healthcare conditions provided to pregnant women [93]. Therefore, in the modern era, the social background and economic condition of the family remain determining factors in pregnancy management and in countering indirect adverse pregnancy outcomes. As part of the SDG, global health-associated organizations have pledged to provide at least a similar healthcare facility to the global population, irrespective of their socioeconomic backgrounds.

Nutritional deficiency and starving

Nutrition is a major factor that directly impacts pregnancy outcomes. Malnutrition remains a cause of huge maternal and neonatal mortality in many LMIC countries. Many African countries have pregnant women in their population who are living 10% beyond the acceptable rate of malnutrition; such a level of malnutrition affects pregnancy outcomes severely even if other factors are managed [94]. Supplementation of vitamins, iron-fortified diets, and other nutritional factors remains the only option to manage such situations [95-97].

Hygiene, sanitation, and altering disease pattern

Since the beginning of obstetrics, hygiene and sanitation have been considered key factors to prevent infection and improve pregnancy outcomes. In the ancient and mediaeval ages, such knowledge was known to us, and proper care was taken. However, with time and environmental conditions, the nature of the infection and the type of pathogen may have changed, other than for some common pathogens. Modern medicine can well manage common pathogenic infections; however, in specific cases, hospital-induced and healthcare-acquired infections may require special attention and the maintenance of proper hygiene and sanitation.

The altered disease pattern can also be a matter of concern. During the earlier pandemics and the recent COVID-19 pandemic, we witnessed the requirement for instant medical preparation, special care, and a novel strategy to manage pregnancy outcomes and reduce maternal morbidity. During the COVID-19 pandemic, previously ignored or unimportant factors such as pandemic containment measures adopted by the country or region and concomitant COVID infection risk to pregnant women became crucial in determining the reason for the pregnancy outcome and maternal mortality [98]. Therefore, with environmental dynamics and special situations such as pandemics, specific strategies and methods should be adopted for maternal care and improved pregnancy outcomes.

Non-communicable diseases, pregnancy, and childbirth

Several studies have been conducted to understand the possible relationship between non-communicable diseases (NCDs) and pregnancy outcomes. In young adults, the possible association of non-communicable diseases with birth by caesarean section suggested no apparent relationship [99]. Early post-natal life and childhood food habits, nutrition, and hygiene maintenance influence the probable susceptibility to NCDs [100,101]. In a study, de Mendonça et al. reported that low birthweight and premature birth were associated with cardiometabolic (CMD) and glycidic metabolism (GMD); further, such birth conditions may also induce the risk of metabolic syndrome during adulthood [102]. Hence, in the absence of any genetic problem, personalized nutrition may help in managing susceptibility to non-communicable diseases during early childhood and adulthood [103].

Environmental exposure and inheritable diseases

Environmental epigenomic conditions influence the disease susceptibility of humans through altering DNA methylation, remodelling chromatin structures, and consecutive gene expression changes [104]. Environmental conditions, especially teratogenic chemical compounds, may impact the foetal epigenome and influence the onset of disease conditions [105]. The likelihood of possible adverse pregnancy outcomes and susceptibility to inheritable genetic diseases was not observed even when the population exposed to nuclear radiation was studied [106]. The influence of the intrauterine environment and outside chemical exposure was suggested to enhance susceptibility to various genetic abnormalities and disease conditions; however, there is a dearth of evidence on the direct association between environmental exposure and inherent genetic conditions.

## Conclusions

The ever-dynamic environmental conditions remain challenging and demanding for global policymakers in relation to reduced maternal and neonatal mortality and better pregnancy outcomes. Since ancient times, it has been a major challenge for medical practitioners and medical caregivers. In recent times, during the COVID-19 pandemic, we have witnessed that sudden alterations in environmental conditions can drastically impact the existing healthcare system and may demand prompt scientific and logical decisions and action to reduce maternal mortality. Therefore, dedicated attention should be provided to maternal healthcare and obstetrics to improve the situation. Furthermore, the present era of global climate change demands more effort and a better strategy apart from the conventional ways to counter the unknown or lesser-known adverse factors associated with maternal and neonatal deaths. More scientific exploration of the recently associated factors may provide information for developing better strategies, policies, and solutions for reducing maternal morbidity, mortality, and neonatal deaths.
